# Imaging the coherent propagation of collective modes in the excitonic insulator Ta_2_NiSe_5_ at room temperature

**DOI:** 10.1126/sciadv.abd6147

**Published:** 2021-07-07

**Authors:** Hope M. Bretscher, Paolo Andrich, Yuta Murakami, Denis Golež, Benjamin Remez, Prachi Telang, Anupam Singh, Luminita Harnagea, Nigel R. Cooper, Andrew J. Millis, Philipp Werner, A. K. Sood, Akshay Rao

**Affiliations:** 1 Cavendish Laboratory, University of Cambridge, Cambridge CB3 0HE, UK.; 2 Department of Physics, Tokyo Institute of Technology, Meguro, Tokyo 152-8551, Japan.; 3 Center for Computational Quantum Physics, Flatiron Institute, New York, NY 10010, USA.; 4 Faculty of Mathematics and Physics, University of Ljubljana, Jadranska 19, SI-1000 Ljubljana, Slovenia.; 5 Jožef Stefan Institute, Jamova 39, SI-1000 Ljubljana, Slovenia.; 6 Department of Physics, Indian Institute of Science Education and Research, Pune, Maharashtra 411008, India.; 7 Department of Physics, Columbia University, New York, NY 10027, USA.; 8 Department of Physics, University of Fribourg, Fribourg 1700, Switzerland.; 9 Department of Physics, Indian Institute of Science, Bangalore, Karnataka 560012, India.

## Abstract

Excitonic insulators host a condensate of electron-hole pairs at equilibrium, giving rise to collective many-body effects. Although several materials have emerged as excitonic insulator candidates, evidence of long-range coherence is lacking and the origin of the ordered phase in these systems remains controversial. Here, using ultrafast pump-probe microscopy, we investigate the possible excitonic insulator Ta_2_NiSe_5_. Below 328 K, we observe the anomalous micrometer-scale propagation of coherent modes at velocities of ~10^5^ m/s, which we attribute to the hybridization between phonon modes and the phase mode of the condensate. We develop a theoretical framework to support this explanation and propose that electronic interactions provide a substantial contribution to the ordered phase in Ta_2_NiSe_5_. These results allow us to understand how the condensate’s collective modes transport energy and interact with other degrees of freedom. Our study provides a unique paradigm for the investigation and manipulation of these properties in strongly correlated materials.

## Teaser

Pump-probe microscopy measurements reveal the presence of rapidly propagating phason-phonon modes in an excitonic condensate.

## INTRODUCTION

**Fig. 1:**
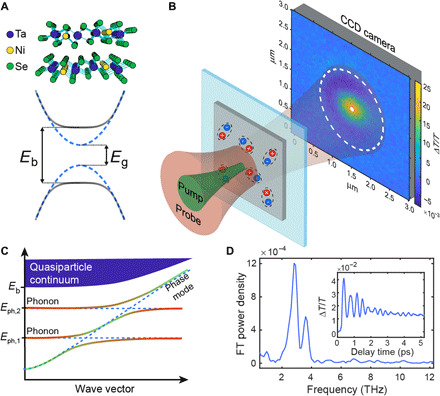
Pump-probe microscopy of Ta_2_NiSe_5_. (**A**) Top: Crystal structure of Ta_2_NiSe_5_. The alternating chains of Ta and Ni atoms confer a quasi-1D nature to the material and independently host conduction and valence band states (*8*). Bottom: Schematic electronic band structure for a prototypical EI. Above the critical temperature (*T*_c_), the material is close to the semiconductor-semimetal transition (portrayed here as a semiconductor of energy gap *E*_g_). Below the *T*_c_, the exciton binding energy (*E*_b_) exceeds the single-particle gap (*E*_g_), resulting in a macroscopic coherent state. (**B**) Schematic of the measurement setup at the location of the sample with the photoinduced transmissivity change (Δ*T*/*T*) signal. The white dot and dashed circle are examples of pixel regions over which we average the signal in our analysis. (**C**) Schematic diagram of a possible low-energy excitation structure for an EI in the presence of electron-phonon coupling. The phonon modes of the material hybridize with the phase mode, resulting in mixed phonon phase modes as long as the energy gap in the phase mode dispersion at *k* = 0 is smaller than the phonon energy *E*_ph,i_. The phonon and phase content of the modes is represented as a color gradient from green (pure phase mode) to red (pure phonon mode). (**D**) Photoinduced transmissivity change (Δ*T*/*T*) as a function of the pump-probe delay time collected at the center of the pump region (inset) and its FT power density.

The excitonic insulator (EI) phase is a state of matter that was first proposed more than 50 years ago (*1*–*3*). It was theorized that below a critical temperature (*T*_c_), weakly screened Coulomb interactions in small bandgap semiconductors and semimetals could lead to the formation of an exciton condensate in the ground state (Fig. 1A). In the proposed theoretical framework, condensation would result from the spontaneous breaking of a U(1) continuous symmetry, which describes the separate conservation of charges in the valence and conduction bands. Therefore, this macroscopic state would be accompanied by characteristic collective excitations, including an amplitude (Higgs) mode and a gapless phase (Goldstone) mode, as is the case for other correlated systems, like superconductors. The emergence of the latter collective mode would lead to intriguing quantum phenomena, such as superfluidic transport (*4*, *5*).

A number of small bandgap or semimetallic transition metal chalcogenide (TMC) compounds have recently attracted interest as promising EI candidates (*6*–*14*). The reduced dimensionality of TMCs results in weak screening of the Coulomb interaction and consequently large exciton binding energies, which could lead to condensation at noncryogenic temperatures. Among this family of materials, particular attention has been devoted to Ta_2_NiSe_5_ following hints of the potential existence of an EI phase below 328 K (*8*–*10*, *12*–*22*). These works have provided insights into the properties in Ta_2_NiSe_5_, for instance, by using equilibrium (*8*, *12*) and time-resolved (*9*, *17*, *18*, *21*, *23*) angle-resolved photoemission spectroscopy (ARPES), by analyzing the effect of physical and chemical pressure (*14*, *22*), by doping (*16*), and by detecting anomalies in charge transport (*14*), optical signatures (*24*, *25*), and phonon properties (*10*, *15*, *19*, *20*, *22*, *26*) as a function of temperature.

However, despite numerous experimental and theoretical studies, the predominant driving mechanism of symmetry breaking in Ta_2_NiSe_5_, and thus the existence and character of the EI phase itself, remains controversial. In real condensed matter systems, contrary to the idealized picture presented above, the Hamiltonian’s continuous symmetry may be further reduced to a discrete symmetry because of electron-phonon interactions or a hybridization between the conduction and valence bands (*27*, *28*). The observed structural phase transition can be driven both by electronic interactions and by electron-phonon coupling (*19*–*21*, *24*, *26*). All these interactions are, in principle, present in a real system, and quantitative differences in their relative magnitudes have a substantial, qualitative impact on the properties of the collective modes in the ordered phase. For instance, the presence of additional symmetry breaking terms in the Hamiltonian forces a gap at *k* ≃ 0 in the dispersion of the phase mode, suppressing the proposed supertransport properties (*29*). Theory suggests that if the transition is largely due to electron-phonon interactions, one would even expect the low-energy, acoustic-like phase mode to be largely suppressed, and an EI phase would not be supported by the material (*28*, *30*). These different pictures could be untangled through the detection of the condensate’s collective modes and the analysis of their transport properties and real-space dynamics. If the phase mode remains gapless or minimally gapped, then the continuous symmetry must remain largely conserved and the transition is predominantly driven by electronic interactions, meaning the system is close to the idealized EI phase.

In this work, we use ultrafast, spatially resolved pump-probe microscopy to study the real-space dynamics of Ta_2_NiSe_5_. We show that photoinduced excitations in Ta_2_NiSe_5_ propagate coherently over distances up to 1 μm and with electron-like velocities, which we attribute to collective modes of the excitonic condensate and their hybridization with the lattice degrees of freedom. These results provide insights toward understanding the nature of the ordered phase in Ta_2_NiSe_5_, potentially establishing a pathway for its manipulation.

## RESULTS

We probe the spatiotemporal dynamics of Ta_2_NiSe_5_ following photoexcitation using a recently developed femtosecond optical pump-probe microscopy technique (*31*–*33*), which provides sub–10-fs time resolution and 10-nm spatial precision (for a characterization of the sample with standard, nonspatially resolved pump-probe spectroscopy, see section S1). In these measurements (see Fig. 1B), we perturb a small area of the sample using a diffraction-limited [≃400-nm full width at half maximum (FWHM)] optical pulse of ≃12-fs duration. We measure the resulting change in transmission using a wide-field (15-μm FWHM), ≃10-fs probe pulse, which is projected onto an electron-multiplying charge-coupled device (EMCCD) camera. The large probe allows us to study both the area directly excited and the surrounding material, imaging how excitations propagate in space and time as the presence of quasiparticles (QPs) or oscillatory modes in the system modulates the probe transmission. Figure 1B displays a schematic of the microscopy setup at the position of the sample and an example of a two-dimensional (2D) plot of the photoinduced transmissivity change (Δ*T*/*T*) as measured 300 fs after the arrival of the pump pulse. Δ*T*/*T* is obtained from (*T*_pump on_ − *T*_pump off_)/(*T*_pump off_), where *T*_pump on_ and *T*_pump off_ indicate the transmission measured in the presence and absence of the pump pulse, respectively. Crucially, as we describe below, we primarily focus our analysis on the regions of the material where we do not directly inject hot QPs.

The kinetic recorded at the center of the excitation spot as a function of the pump-probe delay time is shown in the inset of Fig. 1D and is analogous to those obtained in previous works using nonspatially resolved pump-probe spectroscopy (*10*, *15*). The signal is characterized by an exponentially decaying electronic component superimposed with a strong oscillatory signal. In Fig. 1D, we additionally report the Fourier transform (FT) of this oscillatory component. The main recognizable features are the peaks at 1, 2.9, and 3.6 THz, which have been commonly associated with phonon modes in Ta_2_NiSe_5_ (*10*, *15*).

**Fig. 2:**
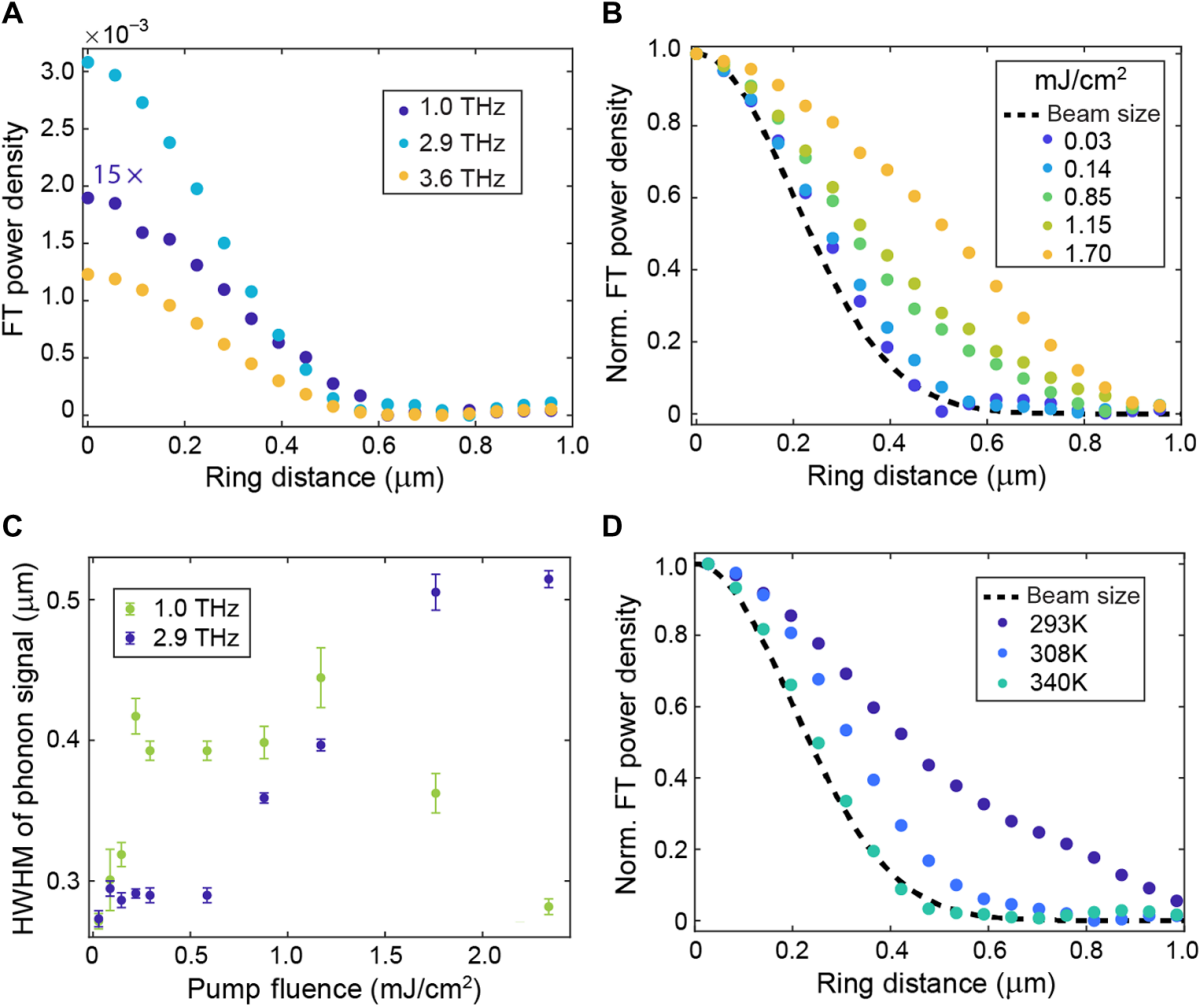
Temperature- and fluence-dependent propagation. (**A**) FT power density as a function of the distance of the ring over which we average the signal from the center of the pump region. These data were collected using a pump fluence of 0.3 mJ/cm^2^. The dataset for the 1-THz mode is magnified by a factor of 15 to improve its visibility. (**B** and **D**) Spatial dependence of the normalized FT power density for the 2.9-THz mode as a function of pump fluence (measured at room temperature) (B) and of temperature (each measured at a comparable fluence of **≃**1.2 mJ/cm^2^) (D). The dashed line indicates the profile of the pump pulse. (**C**) HWHM of the phonon spatial extension as a function of the pump fluence for the 1- and 2.9-THz modes.

The same FT analysis is now applied to the signal in areas away from the pump position, where no excitations are generated directly by the pump pulse. In this case, to focus on coherent propagation out of the photoexcited region and to maximize the signal-to-noise ratio, we average the Δ*T*/*T* signal over rings of pixels equidistant from the center (see section S2 for details on the nearly isotropic nature of the signal). Through this procedure, we determine the spatial decay of the oscillatory modes by plotting the FT power density as a function of the ring distance, as shown in Fig. 2A for the 1-, 2.9-, and 3.6-THz modes. The resulting Gaussian shape is a convolution of the profile of the pump pulse (shown as a reference in Fig. 2, B to D) and of the spatial propagation of the excited modes.

In Fig. 2B, we show the dependence of the FT power density spatial profile for the 2.9-THz mode on the pump fluence. From a Gaussian fit of these curves, we extract the half width at half maximum (HWHM), which is plotted in Fig. 2C. We observe a highly nonlinear dependence of HWHM against the field strength, as the spatial extent of the FT grows after ≃0.55 mJ/cm^2^ and reaches twice its original size before finally saturating when we approach the material breaking point. This result suggests that at low fluences, the signal is dominated by the profile of the pump pulse and the propagation of the mode outside the pumped region becomes visible only at high fluences. While an analogous behavior is observed for the 3.6-THz mode (see section S3), a rather different fluence dependence emerges for the 1-THz mode (Fig. 2C). This result hints at different microscopic mechanisms underlying the excitation of the oscillations, indicating that nonlinear effects may be required to excite the higher-frequency modes.

We next compare (Fig. 2D) the spatial distribution of the FT power density of the 2.9-THz mode above and below *T*_c_ (see section S3 for analogous behavior in the 1- and 3.6-THz modes). While the data are normalized for ease of comparison, the fluence, the overall Δ*T*/*T* signal, and the absolute FT power density at the center of the pumped region are comparable for all these measurements. As *T*_c_ is approached from below, the FT profile narrows. Once the temperature surpasses *T*_c_, the FT profile has shrunk to the size of the pump spot size, suggesting that above *T*_c_, the coherent propagation of the mode breaks down.

**Fig. 3:**
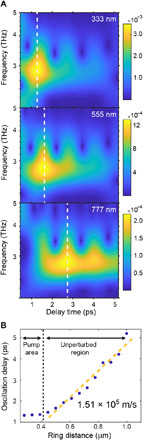
Temporal characterization of the oscillatory modes’ propagation. (**A**) CWT of the oscillatory component of the Δ*T*/*T* signal at three different distances (333, 555, and 777 nm) from the center of the pump region and measured at a fluence of **≃**1.6 mJ/cm^2^. The color map represents the magnitude of the CWT. The white dotted lines approximately mark the location of the strongest phonon oscillations. The periodic oscillations in the CWT are clearly resolved, and their possible origins are discussed in the main text. (**B**) Time delay of the region of strong oscillations as a function of the ring distance. The orange dashed line represents the linear fit of the data in the region outside the pump area and has an inverse slope of 1.51 × 10^5^ m/s.

Returning to room temperature data, we further analyze the time traces for the different rings by performing continuous wavelet transforms (CWTs) (see section S4 for more details). This procedure allows us to determine the amplitude of the oscillatory components as a function of time and distance from the photoexcited area. In Fig. 3A, we show the results of this analysis for a few rings. It can be seen that the strongest amplitude for the 2.9-THz mode (the only one that is clearly traceable within the signal-to-noise limit) progressively shifts toward later times as we move farther away from the pump region. The position of this region in time is represented in Fig. 3B for all the rings. By performing a linear fit of this curve focusing on ring radii residing outside of the directly pumped region, we extract a propagation velocity of 1.51 ± 0.11 × 10^5^ m/s. This means that below *T*_c_, the 2.9-THz mode propagates coherently, at a velocity characteristic of electronic excitations, for distances of up to 1 μm.

## DISCUSSION

We now discuss possible origins of the anomalous spatial propagation at room temperature. First, we note that the measured propagation velocity is orders of magnitude larger than typical values for both optical phonons, which are commonly characterized by rather flat energy dispersions, and acoustic phonons, whose velocity is typically below 10^3^ m/s (*34*). This suggests that the propagating signal does not have a purely phononic origin. Another potential explanation for the observed behavior is the excitation of rapidly propagating bulk or surface phonon-polaritons (*35*, *36*). However, as a result of the crystal inversion symmetry, the bulk phonon modes are Raman and not infrared (IR) active, and therefore cannot participate in the formation of phonon-polariton modes. In addition, for this type of excitation, we would expect to measure higher propagation velocities (>5 × 10^6^ m/s) because of the light-like nature of these modes (see section S8). Last, the disappearance of the propagation above *T*_c_ is unlikely to be explained by this scenario, as the observed phonon modes characterize both the low- and high-temperature phase of this material, which several recent works suggest being an insulator even above *T*_c_ (*25*, *37*). We next consider the possibility of the propagation of QPs emanating from the excitation spot with velocity *v*_k_ = (∂ϵ_k_/∂k) of the order of 10^5^ m/s, which can excite phonons in their wake. However, at room temperature, the carrier relaxation time of QPs is usually only of the order of a few tens of femtoseconds, corresponding to a mean free path of a few nanometers (*38*, *39*). In addition, the emission of phonons by the QPs would generate oscillations that are not in phase as we average rings around the photoexcited region. In our experiments, coherent behavior is observed on a much wider spatial range and on time scales extending to a few picoseconds. We contend that more coherent electronic oscillations (such as the case of plasmons) are unlikely to be excited in this experiment as the material is an insulator at equilibrium. Even after photoexcitation, we therefore do not expect the probed region to host the required carrier population to support these modes. An exciton-polariton, formed by uncondensed excitons at the conduction-band edge, is a final potential candidate that could explain the observed behavior without requiring the existence of any exotic ground state in the system. Yet, long-range and long-lived (several picosecond) excitations, such as the ones that we detect, require considerable coupling between the light and matter modes and substantial confinement. Considering that for comparable van der Waals materials the estimated *Q* factors for a 60-nm flake are ≤5 (*36*), we would not expect to observe modes with lifetimes longer than ∼10 fs in our system (see section S8). Very strong light-matter coupling (resulting in a Rabi splitting of the excitonic line larger than 100 meV) would also be required to achieve experimentally relevant velocities, yet no hints of this are observed in equilibrium measurements (*40*). Last, we are unaware of a mechanism by which these modes could be coherently excited with our pumping scheme, where the injection of QPs with energies much larger than the bandgap will relax to the band edge through successive stochastic relaxation processes. We therefore believe that these considerations make the exciton-polariton scenario rather remote, but analogous measurements in the mid-IR would be useful to more conclusively address this scenario.

One remaining possibility for the anomalous propagation is the coupling between the phonon modes and the EI’s collective phase mode. A phase mode is characterized by a linear dispersion and group velocities on the electronic scale. If electron-phonon coupling is present, the phase and phonon modes can hybridize, resulting in excitations of mixed electronic and phononic nature, which can propagate at velocities close to that of the pure phase mode (see Fig. 1D). The presence of long-range exciton-phonon complexes supporting acoustic-like low-energy excitations is consistent with recent measurements of the optical response functions of Ta_2_NiSe_5_ (*40*, *41*). We expect that the lifetime of the phase mode can be substantially longer than that of QPs, as is supported by a theoretical calculation of the effects of the possible disorder on the QPs and the phase mode [see section S9 and (*42*)]. While the overall relaxation rate of the phonon phase mode might not differ drastically from that of the phonons, the considerably higher speed can result in long propagation lengths (≃ μm) for these excitations. The hybrid modes can then carry a phononic signature ballistically over long distances. This coupled phonon phase mode scenario is also consistent with the suppression of propagation above *T*_c_, where the collective phase mode should disappear. We note that a hybrid phonon phase mode with an electronic-like group velocity is realized as long as the gap of the phase mode is comparable or smaller than the phonon energy. This occurs regardless of the origin of the gap, even where additional electronic terms directly breaking the symmetry (*27*, *28*) also contribute to the formation of the gap.

Returning to Fig. 2C, we can tentatively interpret the different behavior of the two oscillatory modes as the result of a different coupling process between these phonons and the phase mode (see section S6). As the hybridization between the two modes occurs at larger wave vector with increasing phonon frequency, we expect that a higher-order process, like Raman scattering involving multiple phonon modes, could be required to excite the more energetic modes at wave vectors that are not provided by the focused laser beam (see section S5). The saturation and drop in the 1-THz extension at high fluences could be the signature of the enhancement of the gap in the phase mode dispersion. If raised above the phonon energy, the phase mode would not contribute to the formation of the hybrid modes. Further studies that go beyond the scope of this work are required to address these hypotheses.

**Fig. 4:**
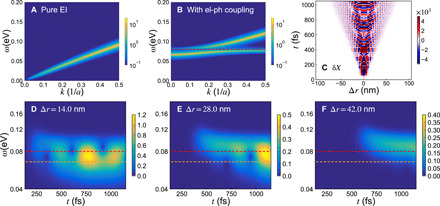
Time-dependent mean-field model. (**A** and **B**) Response function of the order parameter for the microscopic two-band model on the 1D lattice with purely excitonic behavior (A) and in the presence of electron-phonon coupling (B). The coupling is small with respect to the Coulomb interaction and explicitly breaks the continuous symmetry of the system. (**C**) Spatial and temporal evolution of the phonon displacement, δ*X*, for the case of nonzero electron-phonon coupling following an excitation at the center of the system at time *t* = 0, calculated within the mean-field description. (**D** to **F**) Windowed FT of the results in (C) at specified lattice sites, where Δ*r* indicates the distance from the center of the excitation area. The red and orange dashed lines indicate the bare phonon frequency used in these calculations and the size of the phase mode gap, respectively. The bandwidth of the conduction and valence bands is set to 1.6 eV and the Coulomb interaction to 0.84 eV. The corresponding bandgap is about 0.32 eV.

To support our interpretations, we develop a theoretical model within a two-band approximation. We start from a typical Hubbard-type Hamiltonian with additional terms accounting for the electron-phonon coupling and the phonon energy

$$H=H_{\text{kin}}+H_{\text{int}}+H_{\text{el}-\text{ph}}+H_{\text{ph}}$$ (1)

where *H*_kin_ represents the electronic bands, *H*_int_ is the electron-electron interaction, *H*_el−ph_ is the electron-phonon coupling, and *H*_ph_ is the phonon Hamiltonian. This approach is analogous to that used in previous theoretical works on Ta_2_NiSe_5_ (*8*, *37*, *43*), and like there, we use a form of *H*_el−ph_ that explicitly breaks the symmetry of *H* [see section S7 and (*44*) for more details, calculations at various temperatures, and an analysis of the opposite case with no explicit symmetry breaking]. The microscopic parameters corresponding to the band structure and the electron-electron interaction were estimated by fitting previous experimental ARPES results (*8*) (see section S7B). In Fig. 4A, the calculated linear response function shows the dispersion of the massless phase mode in the absence of electron-phonon coupling. Using this dispersion, the estimated group velocity is *v*_PM_ = 1.0 × 10^5^ m/s for this mode, which is of the same order of magnitude of the velocity observed in the experiments. In Fig. 4B, we show the result in the presence of the electron-phonon coupling term, where the phase mode becomes massive and a hybridization between the phase and phonon mode occurs.

To determine whether the phonon oscillations induced by the excitation can propagate at velocities of the order of *v*_PM_ (an electronic-like velocity), we perform a real-space time-dependent mean-field simulation (see section 7A). Here, we use a larger phonon frequency (indicated by the red dashed line in Fig. 4, B and D to F) than those observed in the experimental data, and we correspondingly consider a smaller excitation area. These assumptions do not affect the conclusions that we can draw from these calculations, and they are adopted only for the sake of simplifying the numerical simulation. In Fig. 4C, we show the propagation of the phonon displacement after a spatially confined excitation. The signal propagates with a velocity of about 0.6 × 10^5^ m/s. We can visualize the time evolution of the phonon displacement at different distances from the excitation by performing a spatially windowed FT, analogous to a CWT analysis. The results are shown in Fig. 4 (D to F), where the orange dashed line indicates the frequency associated with the gap in the phase mode dispersion for the parameters used in our simulations. These plots illustrate the spreading of the hybrid mode at the phonon frequency with a velocity comparable to the phase mode velocity and further support the above scenario that mixing between the phase mode and the phonon mode leads to the fast propagation of the phonon oscillations.

We note that in Fig. 4 (D to F), oscillations in the phonon displacement as a function of time contain information about the gap of the phase mode at *k* ≃ 0 (see section S7B for details). While the data in Fig. 3A show analogous oscillations, we presently cannot determine whether their origin is connected to the gapped phase mode or to a more trivial frequency beating between the 2.9- and 3.6-THz modes.

Using ultrafast pump-probe microscopy, we image the spatial dynamics of oscillatory modes in the EI candidate Ta_2_NiSe_5_. Below 328 K, we observe the anomalous propagation of these modes, which remain coherent up to ≃1 μm at a velocity of 1.5 × 10^5^ m/s. After carefully considering various scenarios, we attribute this propagation to the hybridization of dispersionless phonon modes with the dispersive, low-lying phase mode of an excitonic condensate, which are excited through the abrupt change in the QP density in the excitation region (*45*). Further studies are required to establish the precise processes by which these collective modes are excited. Our results and interpretations have several implications. First, the experimental observation of the signature of a phase mode provides us with important insight into the nature of the excitonic order in Ta_2_NiSe_5_. Namely, if the gap of the phase mode was much larger than the phonon frequency, the mixing between the modes would be suppressed. Hence, the effects of the electron-phonon coupling and of possible electronic terms that explicitly break the continuous symmetry (*27*–*29*, *43*) should be weak, indicating that the ordered phase in Ta_2_NiSe_5_ is an EI phase primarily driven by interband Coulomb interactions (see section S7). Second, a notable experimental observation is the small anisotropy in the mode propagation. This may seem counterintuitive because Ta_2_NiSe_5_ is a quasi-1D system, and previous density functional theory calculations found that the bandwidth of the valence band along the direction perpendicular to the atomic chains should be almost five times smaller than in the direction along the chains (*13*). However, we show theoretically that even in the presence of anisotropic hopping between the lattice sites, when the system approaches the Bardeen-Cooper-Schrieffer (BCS)–Bose-Einstein condensation (BEC) crossover regime, the anisotropy of the phase mode velocity becomes small (see section S7B3). Hence, the small experimentally observed anisotropy indicates that the system is close to the BCS-BEC crossover regime, as suggested in a previous work (*14*). Last, our results showcase the possibility of using spatially resolved, femtosecond pump-probe measurements to gain crucial insights into the properties of condensed matter systems manifesting emergent many-body phenomena. By exciting and detecting coherent collective modes and by studying their propagation properties, important information can be gathered on the microscopic origin of these effects. Femtosecond pulses could thus provide a means to control and read out the properties of these systems in future quantum information applications.

## MATERIALS AND METHODS

### Sample preparation

Ta_2_NiSe_5_ crystals were grown using the procedure outlined in (*22*). To prepare samples for optical measurements, flakes were exfoliated using gold-assisted exfoliation (*46*) onto glass coverslips and subsequently encapsulated with a second glass slide while inside a nitrogen glovebox.

### Pump-probe microscopy

A Yb:KGW laser system (Pharos, Light Conversion) delivers 1030-nm light with pulses of 200-fs duration and 30-μJ power at a repetition rate of 200 kHz. The output beam is split to seed two broadband white-light continuum generation stages used to create pump and probe pulses. The broadband probe beam is generated using a 3-mm yttrium-aluminum-garnet (YAG) crystal, and a fused-silica prism is then used to select the spectral range of interest (from 650 to 950 nm). The white-light continuum used for pump pulses is instead generated using a 3-mm sapphire crystal, delivering pulses covering the wavelength range from 500 to 650 nm [a long wavelength cutoff is achieved using a short-pass filter (FESH650, Thorlabs)]. The pulses are temporally compressed using chirped mirrors and fused silica wedges to achieve <12-fs pulses. In particular, two sets of Layertec (109811) mirrors are used on the pump path to also compensate for the glass elements contained in the objective; Venteon (DCM9) (Venteon) mirrors are instead used in the probe path. The pulse length is verified using a frequency-resolved optical gating technique (*47*). A mechanical chopper (Thorlabs, MC2000B) is used to modulate the pump pulse stream at a frequency of 30 Hz, and a 40-μm pinhole is used to spatially clean the laser mode. The pump beam is finally expanded to achieve proper filling of the back aperture of an oil-immersion, 1.1 numerical aperture (NA) objective, which finally focuses the pulses onto the sample to a spot size of ∼400-nm FWHM. Reflected components of the pump are removed from the collection path using a long-pass filter. The probe pulses are mechanically delayed with respect to the pump excitation using a closed-loop piezo translation stage (P-625.1CL, Physik Instrumente) and then focused onto the sample to a size of ∼15-μm FWHM using a spherical mirror. The transmitted probe pulse is collected by the objective, sent through a 10-nm bandpass filter (Thorlabs) to select the wavelength range of interest, and finally imaged onto an EMCCD camera (Rolera Thunder, QImaging). For further details on the experimental apparatus, see (*32*).

## SUPPLEMENTARY MATERIALS

Supplementary material for this article is available at http://advances.sciencemag.org/cgi/content/full/7/28/eabd6147/DC1

## References

[1] D. Jérome, T. M. Rice, W. Kohn, Excitonic insulator. Phys. Rev. 158, 462–475 (1967).

[2] B. I. Halperin, T. M. Rice, Possible anomalies at a semimetal-semiconductor transistion. Rev. Mod. Phys. 40, 755–766 (1968).

[3] L. V. Keldysh, A. N. Kozlov, Collective properties of excitons in semiconductors. Sov. Phys. JETP 27, 521 (1968).

[4] P. Coleman, *Introduction to Many-Body Physics* (Cambridge Univ. Press, 2015).

[5] E. Hanamura, H. Haug, Will a bose-condensed exciton gas be superfluid? Solid State Commun. 15, 1567–1570 (1974).

[6] H. Cercellier, C. Monney, F. Clerc, C. Battaglia, L. Despont, M. G. Garnier, H. Beck, P. Aebi, L. Patthey, H. Berger, L. Forró, Evidence for an excitonic insulator phase in 1*T*‑TiSe_2_. Phys. Rev. Lett. 99, 146403 (2007). 17930692 10.1103/PhysRevLett.99.146403

[7] C. Monney, C. Battaglia, H. Cercellier, P. Aebi, H. Beck, Exciton condensation driving the periodic lattice distortion of 1T-TiSe_2_. Phys. Rev. Lett. 106, 106404 (2011). 21469817 10.1103/PhysRevLett.106.106404

[8] K. Seki, Y. Wakisaka, T. Kaneko, T. Toriyama, T. Konishi, T. Sudayama, N. L. Saini, M. Arita, H. Namatame, M. Taniguchi, N. Katayama, M. Nohara, H. Takagi, T. Mizokawa, Y. Ohta, Excitonic Bose-Einstein condensation in Ta_2_NiSe_5_ above room temperature. Phys. Rev. B 90, 155116 (2014).

[9] S. Mor, M. Herzog, D. Golež, P. Werner, M. Eckstein, N. Katayama, M. Nohara, H. Takagi, T. Mizokawa, C. Monney, J. Stähler, Ultrafast electronic band gap control in an excitonic insulator. Phys. Rev. Lett. 119, 086401 (2017). 28952776 10.1103/PhysRevLett.119.086401

[10] D. Werdehausen, T. Takayama, M. Höppner, G. Albrecht, A. W. Rost, Y. Lu, D. Manske, H. Takagi, S. Kaiser, Coherent order parameter oscillations in the ground state of the excitonic insulator Ta_2_NiSe_5_. Sci. Adv. 4, eaap8652 (2018). 29740599 10.1126/sciadv.aap8652 PMC5938280

[11] A. Kogar, M. S. Rak, S. Vig, A. A. Husain, F. Flicker, Y. I. Joe, L. Venema, G. J. MacDougall, T. C. Chiang, E. Fradkin, J. van Wezel, P. Abbamonte, Signatures of exciton condensation in a transition metal dichalcogenide. Science 358, 1314–1317 (2017). 29217574 10.1126/science.aam6432

[12] Y. Wakisaka, T. Sudayama, K. Takubo, T. Mizokawa, M. Arita, H. Namatame, M. Taniguchi, N. Katayama, M. Nohara, H. Takagi, Excitonic insulator state in Ta_2_NiSe_5_ probed by photoemission spectroscopy. Phys. Rev. Lett. 103, 026402 (2009). 19659224 10.1103/PhysRevLett.103.026402

[13] T. Kaneko, T. Toriyama, T. Konishi, Y. Ohta, Orthorhombic-to-monoclinic phase transition of Ta_2_NiSe_5_ induced by the Bose-Einstein condensation of excitons. Phys. Rev. B 87, 035121 (2013).

[14] Y. F. Lu, H. Kono, T. I. Larkin, A. W. Rost, T. Takayama, A. V. Boris, B. Keimer, H. Takagi, Zero-gap semiconductor to excitonic insulator transition in Ta_2_NiSe_5_. Nat. Commun. 8, 14408 (2017). 28205553 10.1038/ncomms14408 PMC5316885

[15] S. Mor, M. Herzog, J. Noack, N. Katayama, M. Nohara, H. Takagi, A. Trunschke, T. Mizokawa, C. Monney, J. Stähler, Inhibition of the photoinduced structural phase transition in the excitonic insulator Ta_2_NiSe_5_. Phys. Rev. B 97, 115154 (2018).

[16] L. Chen, T. T. Han, C. Cai, Z. G. Wang, Y. D. Wang, Z. M. Xin, Y. Zhang, Doping-controlled transition from excitonic insulator to semimetal in Ta_2_NiSe_5_. Phys. Rev. B 102, 161116 (2020).

[17] T. Suzuki, Y. Shinohara, Y. Lu, M. Watanabe, J. Xu, K. L. Ishikawa, H. Takagi, M. Nohara, N. Katayama, H. Sawa, M. Fujisawa, T. Kanai, J. Itatani, T. Mizokawa, S. Shin, K. Okazaki, Detecting electron-phonon couplings during photo-induced phase transition. Phys. Rev. B 103, 121105 (2021).

[18] T. Tang, H. Wang, S. Duan, Y. Yang, C. Huang, Y. Guo, D. Qian, W. Zhang, Non-coulomb strong electron-hole binding in Ta_2_NiSe_5_ revealed by time- and angle-resolved photoemission spectroscopy. Phys. Rev. B 101, 235148 (2020).

[19] K. F. Kim, H. Kim, J. Kim, C. Kwon, J. S. Kim, B. J. Kim, Direct observation of excitonic instability in Ta_2_NiSe_5_. Nat. Commun. 12, 1969 (2021). 33785740 10.1038/s41467-021-22133-z PMC8010035

[20] P. A. Volkov, M. Ye, H. Lohani, I. Feldman, A. Kanigel, G. Blumberg, Critical charge fluctuations and quantum coherent state in excitonic insulator Ta_2_NiSe_5_. npj Quant. Mater. 6, 52 (2021).

[21] T. Saha, D. Golez, G. De Ninno, J. Mravlje, Y. Murakami, B. Ressel, M. Stupar, P. R. Ribic, Photo-induced phase transition and associated time scales in the excitonic insulator Ta_2_NiSe_5_. Phys. Rev. B 103, 144304 (2021).

[22] S. Pal, S. Grover, L. Harnagea, P. Telang, A. Singh, D. V. S. Muthu, U. V. Waghmare, A. K. Sood, Destabilizing excitonic insulator phase by pressure tuning of exciton-phonon coupling. Phys. Rev. Res. 2, 043182 (2020).

[23] K. Okazaki, Y. Ogawa, T. Suzuki, T. Yamamoto, T. Someya, S. Michimae, M. Watanabe, Y. Lu, M. Nohara, H. Takagi, N. Katayama, H. Sawa, M. Fujisawa, T. Kanai, N. Ishii, J. Itatani, T. Mizokawa, S. Shin, Photo-induced semimetallic states realised in electron-hole coupled insulators. Nat. Commun. 9, 4322 (2018). 30333495 10.1038/s41467-018-06801-1 PMC6192982

[24] H. Ning, O. Mehio, M. Buchhold, T. Kurumaji, G. Refael, J. G. Checkelsky, D. Hsieh, Signatures of ultrafast reversal of excitonic order in Ta_2_NiSe_5_. Phys. Rev. Lett. 125, 267602 (2020). 33449742 10.1103/PhysRevLett.125.267602

[25] H. M. Bretscher, P. Andrich, P. Telang, A. Singh, L. Harnaga, A. K. Sood, A. Rao, Ultrafast melting and recovery of collective order in the excitonic insulator Ta_2_NiSe_5_. Nat. Commun. 12, 1699 (2021). 33727541 10.1038/s41467-021-21929-3 PMC7966769

[26] M.-J. Kim, A. Schulz, T. Takayama, M. Isobe, H. Takagi, S. Kaiser, Phononic soft mode behavior and a strong electronic background across the structural phase transition in the excitonic insulator Ta_2_NiSe_5_. Phys. Rev. Res. 2, 042039 (2020).

[27] G. Mazza, M. Rösner, L. Windgätter, S. Latini, H. Hübener, A. J. Millis, A. Rubio, A. Geroges, Nature of symmetry breaking at the excitonic insulator transition: Ta_2_NiSe_5_. Phys. Rev. Lett. 124, 197601 (2020). 32469559 10.1103/PhysRevLett.124.197601

[28] M. D. Watson, I. Marković, E. A. Morales, P. Le Fèvre, M. Merz, A. A. Haghighirad, P. D. C. King, Band hybridisation at the semimetal-semiconductor transition of Ta_2_NiSe_5_ enabled by mirror-symmetry breaking. Phys. Rev. Res. 2, 013236 (2020).

[29] B. Zenker, H. Fehske, H. Beck, Fate of the excitonic insulator in the presence of phonons. Phys. Rev. B 90, 195118 (2014).

[30] E. Baldini, A. Zong, D. Choi, C. Lee, M. H. Michael, L. Windgaetter, I. I. Mazin, S. Latini, D. Azoury, B. Lv, A. Kogar, Y. Wang, Y. Lu, T. Takayama, H. Takagi, A. J. Millis, A. Rubio, E. Demler, N. Gedik, The spontaneous symmetry breaking in Ta_2_NiSe_5_ is structural in nature. arXiv:2007.02909 [cond-mat.str-el] (6 July 2020). 10.1073/pnas.2221688120 PMC10151608 37071679

[31] G. V. Hartland, Ultrafast studies of single semiconductor and metal nanostructures through transient absorption microscopy. Chem. Sci. 1, 303–309 (2010).

[32] C. Schnedermann, J. Sung, R. Pandya, S. D. Verma, R. Y. S. Chen, N. Gauriot, H. M. Bretscher, P. Kukura, A. Rao, Ultrafast tracking of exciton and charge carrier transport in optoelectronic materials on the nanometer scale. J. Phys. Chem. Lett. 10, 6727–6733 (2019). 31592672 10.1021/acs.jpclett.9b02437 PMC6844127

[33] J. Sung, C. Schnedermann, L. Ni, A. Sadhanala, R. Y. S. Chen, C. Cho, L. Priest, J. M. Lim, H.-K. Kim, B. Monserrat, P. Kukura, A. Rao, Long-range ballistic propagation of carriers in methylammonium lead iodide perovskite thin films. Nat. Phys. 16, 171–176 (2020).

[34] V. S. Gorelik, N. S. Vasil’ev, Dispersion of optical and acoustic phonons in diamond and germanium crystals. Inorg. Mater. 48, 462–468 (2012).

[35] S. Dai, Z. Fei, Q. Ma, A. S. Rodin, M. Wagner, A. S. McLeod, M. K. Liu, W. Gannett, W. Regan, K. Watanabe, T. Taniguchi, M. Thiemens, G. Dominguez, A. H. C. Neto, A. Zettl, F. Keilmann, P. Jarillo-Herrero, M. M. Fogler, D. N. Basov, Tunable phonon polaritons in atomically thin van der *w*aals crystals of boron nitride. Science 343, 1125–1129 (2014). 24604197 10.1126/science.1246833

[36] D. N. Basov, M. M. Fogler, F. J. G. De Abajo, Polaritons in van der Waals materials. Science 354, aag1992 (2016). 27738142 10.1126/science.aag1992

[37] K. Sugimoto, S. Nishimoto, T. Kaneko, Y. Ohta, Strong coupling nature of the excitonic insulator state in Ta_2_NiSe_5_. Phys. Rev. Lett. 120, 247602 (2018). 29956960 10.1103/PhysRevLett.120.247602

[38] D. Gall, Electron mean free path in elemental metals. J. Appl. Phys. 119, 085101 (2016).

[39] A. Jablonski, P. Mrozek, G. Gergely, M. Menhyard, A. Sulyok, The inelastic mean free path of electrons in some semiconductor compounds and metals. Surf. Interface Anal. 6, 291–294 (1984).

[40] T. I. Larkin, A. N. Yaresko, D. Pröpper, K. A. Kikoin, Y. F. Lu, T. Takayama, Y.-L. Mathis, A. W. Rost, H. Takagi, B. Keimer, A. V. Boris, Giant exciton fano resonance in quasi-one-dimensional Ta_2_NiSe_5_. Phys. Rev. B 95, 195144 (2017).

[41] T. I. Larkin, R. D. Dawson, M. Höppner, T. Takayama, M. Isobe, Y.-L. Mathis, H. Takagi, B. Keimer, A. V. Boris, Infrared phonon spectra of quasi-one-dimensional Ta_2_NiSe_5_ and Ta_2_NiS_5_. Phys. Rev. B 98, 125113 (2018).

[42] B. Remez, N. R. Cooper, Effects of disorder on the transport of collective modes in an excitonic condensate. Phys. Rev. B 101, 235129 (2020).

[43] Y. Murakami, D. Golež, M. Eckstein, P. Werner, Photoinduced enhancement of excitonic order. Phys. Rev. Lett. 119, 247601 (2017). 29286755 10.1103/PhysRevLett.119.247601

[44] Y. Murakami, D. Golež, T. Kaneko, A. Koga, A. J. Millis, P. Werner, Collective modes in excitonic insulators: Effects of electron-phonon coupling and signatures in the optical response. Phys. Rev. B 101, 195118 (2020).

[45] T. K. Cheng, J. Vidal, H. J. Zeiger, G. D. M. S. Dresselhaus, M. S. Dresselhaus, E. P. Ippen, Mechanism for displacive excitation of coherent phonons in Sb, Bi, Te, and Ti_2_O_3_. Appl. Phys. Lett. 59, 1923–1925 (1991).

[46] S. B. Desai, S. R. Madhvapathy, M. Amani, D. Kiriya, M. Hettick, M. Tosun, Y. Zhou, M. Dubey, J. W. Ager III, D. Chrzan, A. Javey, Gold-mediated exfoliation of ultralarge optoelectronically perfect monolayers. Adv. Mater. 28, 4053–4058 (2016). 27007751 10.1002/adma.201506171

[47] R. Trebino, K. W. De Long, D. N. Fittinghoff, J. N. Sweetser, M. A. Krumbügel, B. A. Richman, D. J. Kane, Measuring ultrashort laser pulses in the time-frequency domain using frequency-resolved optical gating. Rev. Sci. Instrum. 68, 3277–3295 (1997).

[48] H. L. Stern, A. Cheminal, S. R. Yost, K. Broch, S. L. Bayliss, K. Chen, M. Tabachnyk, K. Thorley, N. Greenham, J. M. Hodgkiss, J. Anthony, M. Head-Gordon, A. J. Musser, A. Rao, R. H. Friend, Vibronically coherent ultrafast triplet-pair formation and subsequent thermally activated dissociation control efficient endothermic singlet fission. Nat. Chem. 9, 1205–1212 (2017). 29168494 10.1038/nchem.2856

[49] A. Dubietis, G. Tamošauskas, R. Šuminas, V. Jukna, A. Couairon, Ultrafast supercontinuum generation in bulk condensed media (Invited Review). Lith. J. Phys. 53, 113 (2017).

[50] J. D. Caldwell, A. V. Kretinin, Y. Chen, V. Giannini, M. M. Fogler, Y. Francescato, C. T. Ellis, J. G. Tischler, C. R. Woods, A. J. Giles, M. Hong, K. Watanabe, T. Taniguchi, S. A. Maier, K. S. Novoselov, Sub-diffractional volume-confined polaritons in the natural hyperbolic material hexagonal boron nitride. Nat. Commun. 5, 5221 (2014). 25323633 10.1038/ncomms6221

[51] Z. Fei, M. E. Scott, D. J. Gosztola, J. J. Foley IV, J. Yan, D. G. Mandrus, H. Wen, P. Zhou, D. W. Zhang, Y. Sun, J. R. Guest, S. K. Gray, W. Bao, G. P. Wiederrecht, X. Xu, Nano-optical imaging of WSe_2_ waveguide modes revealing light-exciton interactions. Phys. Rev. B 94, 081402 (2016).

[52] G. D. Mahan, *Many-Particle Physics* (Springer Science & Business Media, 2013).

[53] M. Mrejen, L. Yadgarov, A. Levanon, H. Suchowski, Transient exciton-polariton dynamics in WSe_2_ by ultrafast near-field imaging. Sci. Adv. 5, eaat9618 (2019). 30746484 10.1126/sciadv.aat9618 PMC6358311

[54] A. L. Fetter, J. D. Welecka, *Quantum Theory of Many-Particle System* (Dover, 2003).

